# Complete Genome Sequence of a Polish Enterococcus faecalis *vanA*-Positive Hospital Isolate

**DOI:** 10.1128/MRA.00668-21

**Published:** 2021-10-07

**Authors:** Ewa Wardal, Ewa Sadowy

**Affiliations:** a Department of Molecular Microbiology, National Medicines Institute, Warsaw, Poland; Loyola University Chicago

## Abstract

Enterococcus faecalis is an important human pathogen involved in health care-associated infections, and its increasing resistance to vancomycin is worrisome. Here, we report the complete genome sequence of a Polish hospital *vanA-*positive isolate of E. faecalis, consisting of a 3,264,821-bp chromosome and six plasmids.

## ANNOUNCEMENT

Enterococcus faecalis plays a significant role in health care-associated infections (HAIs), causing both invasive (bacteremia, infective endocarditis) and noninvasive (urinary tract, wound) infections (1). E. faecalis treatment options are significantly limited, due to its intrinsic and acquired resistance to several antimicrobials (2). An acquisition of resistance to glycopeptides (vancomycin and teicoplanin) by clinical enterococci is typically associated with the presence of *vanA* or *vanB* gene clusters (3) and further limits successful therapy. Here, we present the complete sequence of the genome of a hospital *vanA-*positive isolate of E. faecalis obtained in Poland.

E. faecalis isolate 1207/14, recovered from the stool of a patient hospitalized in Ostrów Mazowiecka, Poland (52.8071 N, 21.8796 E), was received in 2014 by the National Reference Centre for Susceptibility Testing (NRCST). The isolate was obtained during routine national surveillance activity of the NRCST, under the mandate of the Ministry of Health. The study was performed in a retrospective manner with anonymization of the patient’s data; thus, ethical approval and informed consent were not required.

The isolate was streaked for single colonies onto sheep blood (SB) agar plates and incubated at 37°C for 18 h. A single colony was transferred into tryptic soy broth (TSB) liquid medium, incubated for 18 h, and frozen in 10% glycerol at −80°C as part of the NRCST lab culture collection. A sample of frozen stock was streaked onto SB agar plates and grown overnight at 37°C; bacterial cultures were collected, and a genomic DNA Prep Plus kit (A&A Biotechnology, Gdansk, Poland) was used for genomic DNA extraction for Illumina and Nanopore sequencing, following the manufacturer’s protocol.

Genomic DNA was sequenced by Genomed S.A. (Warsaw, Poland) on the MiSeq platform (Illumina Inc., San Diego, CA) in PE300 mode. The workflow used a NEBNext DNA library prep master mix set for Illumina (NEB) for library generation. Sequencing generated 668,546150-bp paired-end reads, which were trimmed using Cutadapt version 1.16 (4), yielding a total of 333,903 paired reads and 159.3 Mbp of sequencing data.

A separate sample of genomic DNA was sequenced by Genomed S.A. (Warsaw, Poland) on the MinION device (Oxford Nanopore Technologies, UK) using a FLO-MIN111 (R10.3) flow cell with 2 μg of DNA, prepared using the ligation sequencing kit 1D (SQK-LSK109) without shearing. Reads were base called during sequencing using Guppy version 4.0.15, generating 189,000 reads and a total of 1,064.0 Mbp of sequencing data (*N*_50_, 11,469 bp).

Hybrid assembly of the Illumina and MinION reads (total coverage, 400×) was performed using Unicycler version 0.4.7 (5) and resulted in a single chromosome and six plasmids (Table 1). These chromosome and plasmid sequences were annotated using NCBI PGAP version 4.13 (8) and were predicted to contain 3,500 genes, including 3,327 protein-coding sequences, 4 complete sets of rRNAs (5S, 16S, and 23S rRNAs), 70 tRNAs, four noncoding RNAs (ncRNAs), and 87 pseudogenes. The *vanA* operon is located on plasmid p1207_4. Additionally, resistance genes to aminoglycosides, tetracycline, macrolides/lincosamides/streptogramin B, and chloramphenicol are present in the genome of E. faecalis isolate 1207/14 (Table 1). In multilocus sequence typing (MLST) (9), this isolate represents sequence type 464, as established using the PubMLST database (https://pubmlst.org/organisms/enterococcus-faecalis) (10).

**TABLE 1:** Characteristics of the genome of E. faecalis isolate 1207/14

Contig	%GC	Size (bp)	No. of CDSs^*a*^	GenBank accession no.	AMR gene(s)^*b*^	Plasmid replicon type^*c*^
Chromosome	37.2	3,264,821	3,163	CP075604.1	*aac(6′)-aph(2″) tet*(M)	NA
p1207_1	35.0	75,084	91	CP075605.1	*cat*_pC221_, *erm*(B), *aph(3′)-III*, *ant(6)-Ia*	*rep9b*_pEF62pC_, *rep6*_pS86_, *rep7a*_pRE25_, *repUS12*_SAP014A_
p1207_2	34.0	70,628	76	CP075606.1	None	*repUS11*_pTEF3_
p1207_3	34.5	46,461	50	CP075607.1	None	*rep9a*_pAD1_
p1207_4	35.0	21,607	24	CP075608.1	*vanA* operon	*repUS1*_pVEF1_, *rep18a*_p200B_, *repUS12*_SAP014A_
p1207_5	36.5	4,375	6	CP075609.1	None	*rep14a*_AUS0085p5_
p1207_6	37.8	2,056	4	CP075610.1	None	Unknown

a CDSs, coding DNA sequences.

b Established using ResFinder (https://cge.cbs.dtu.dk/services/ResFinder/) (6).

c Established using PlasmidFinder (https://cge.cbs.dtu.dk/services/PlasmidFinder/) (7). NA, not applicable.

Default parameters were used for all software.

### Data availability.

This genome project is indexed at GenBank under BioProject accession number PRJNA731638. The complete genome sequence for Enterococcus faecalis isolate 1207/14 can be found under GenBank accession number CP075604 for the chromosome and CP075605 to CP075610 for plasmids p1207_1 through p1207_6 (Table 1). The Illumina reads can be found under SRA accession number SRR14817168. Fastq files from the MinION run can be found under SRA run accession number SRR14861606. A log file generated by Unicycler for assembly of this genome sequence can be found at figshare (https://doi.org/10.6084/m9.figshare.14806221).
